# Whole transcriptome data of zebrafish exposed to chronic dose of depleted uranium

**DOI:** 10.1016/j.dib.2017.07.071

**Published:** 2017-07-28

**Authors:** Olivier Armant, Kewin Gombeau, Christelle Adam Guillermin

**Affiliations:** Institut de Radioprotection et de Sureté Nucléaire (IRSN), PRP-ENV/SERIS/LECO, Cadarache, Saint-Paul-lez-Durance 13115, France

**Keywords:** Genetics, mRNASeq, Zebrafish, Depleted uranium, Development, Multigenerational effects

## Abstract

The concentration of depleted uranium (DU) in the environment is expected to increase due to anthropogenic activities, posing potential risks on ecosystems. The effects of chronic exposure to DU at concentration close to the environmental standards (0.3–30 µg DU/L) are scarcely characterised. Genomic alterations caused by low doses of pollutants can potentially propagate over generations, but how these effects may affect the health of the progeny remain uncertain for the vast majority of toxicants. The present dataset describes the transcriptomic effects of a chronic exposure to 20 µg DU/L during 10 days on adult zebrafish (*Danio rerio*) organs, the brain, the testis and the ovaries. The potential multigenerational effects of DU were assessed on the progeny of the adult exposed fish at the two-cells stage and after four days of development. We describe in this article the summary statistics of the differential gene expression analysis and focus on key molecular pathways affected by an exposure to a low concentration of DU. The data presented in this study supports the observation made in Armant et al. (2017) [1] (https://doi.org/10.1016/j.dib.2016.05.007) that DU can induce a molecular stress in both adult zebrafish and their progeny. The raw dataset has been deposited at the Gene Expression Omnibus (GEO) repository under the accession number *GEO:*GSE96603.

**Specification Table**

**Table:** 

Subject area	*Biology*
More specific subject area	*Bioinformatics and toxicogenomics*
Type of data	*Figures, tables*
How data was acquired	*High-throughput RNA sequencing*
Data format	*Filtered and analysed with statistical tests*
Experimental factors	*Wild type versus exposed to depleted uranium*
Experimental features	*Comparison of the transcriptomic response from adult zebrafish tissues (brain, ovaries and testis) exposed to depleted uranium and their progeny (at two times of development) to their respective controls. Triplicates were used for each condition. Directional libraries were sequenced on Illumina HiSeq. 15000 in paired-end reads*
Data source location	*Institut de Radioprotection et de Sureté Nucléaire (IRSN), PRP-ENV/SERIS/LECO, Cadarache, Saint-Paul-lez-Durance 13115, France.*
Data accessibility	*Data are available with this article, and via NCBI*′*s GEO accession number GEO:*GSE96603http://www.ncbi.nlm.nih.gov/geo/query/acc.cgi?acc=GSE96603

**Value of the data**

• Depleted uranium is a heavy metal posing potential environmental risks due to its increasing release from anthropogenic activities.
• This dataset presents the differentially expressed genes in adult brain and gonads (testis and ovaries) from zebrafish exposed to 20 µg/L depleted uranium for 10 days.
• It also provides the potential multigenerational effects of a parental exposure to depleted uranium in the progeny of exposed fish at both the two-cells stage and on four-days larvae.
• The analysis of the biological pathways impacted by a chronic depleted uranium exposure will help to understand the molecular mechanisms of toxicity of this toxicant or other heavy metals.
• The identification of the depleted uranium (DU) de-regulated genes could lead to the development of biomarkers of DU and other heavy metals.

## 1 Data

**Fig. 1:**
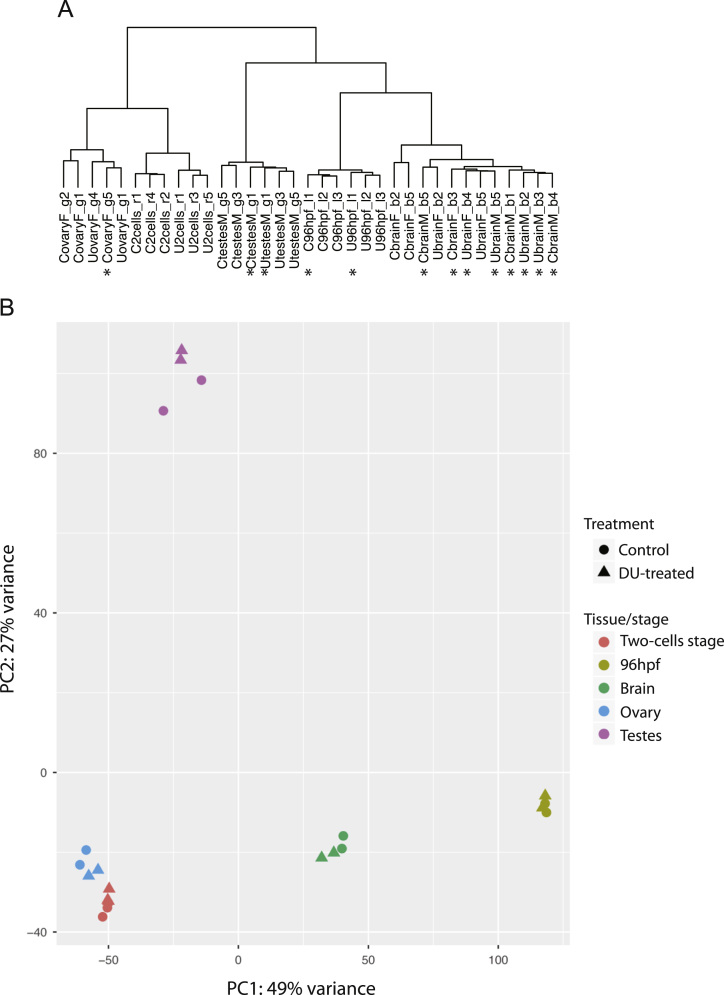
Correlation of biological replicates. A. Dendrogram of the 35 samples normalized using the regularized log transformation method from DESeq. 2 using Spearman's correlation and the absolute linkage method. The samples marked by * have a higher divergence compared to the other replicates C: control, U: DU-treated, M: male, F: female. The stage/condition and the replicate number are indicated for each sample. B. Principal component analysis on 22 samples with low biological variabilities. Stage and tissues are indicated by the colour code. Round-shape correspond to controls and triangle to DU-exposed samples.

**Fig. 2:**
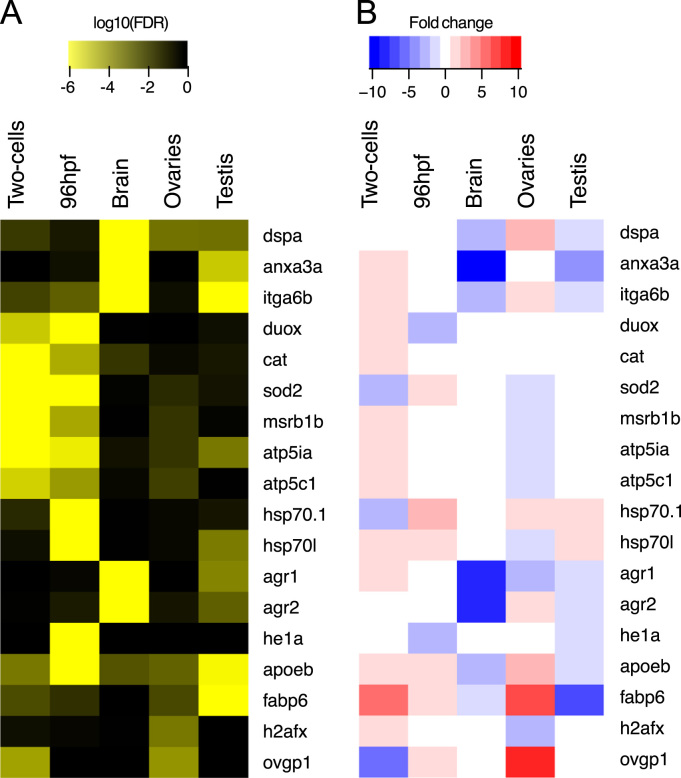
Heatmap of a selection of differentially expressed genes. A. Significant FDR from each comparative analysis are displayed in yellow (depleted uranium versus control), and non-significant FDR in black. Absent FDR were set to 1 (non-significant) and FDR<10^−6^ fitted to 10^−6^. B. Fold changes obtained from the differential gene expression analysis: down-regulated genes in blue, no change in expression in white, up-regulated genes in red.

This data consists of 35 high-throughput sequencing samples of adult brain, testis and ovaries obtained from adult zebrafish exposed to 20 µg/L of depleted uranium (DU) for 10 days, as well as their progeny both at the two-cells stage and four-days larvae (96 h post-fertilization, hpf) [1], [2]. The data are deposited under the Gene Expression Omnibus (GEO) number *GEO:* GSE96603 at http://www.ncbi.nlm.nih.gov/geo/query/acc.cgi?acc=GSE96603. The list of samples collected in this study is provided in Table 1. The principal component analysis on the regularized log transformed (rlog) expression data shows at the global level that the biological replicates group by stage and tissue (Fig. 1). A selection of 22 samples with low biological variability was made for the differential expression analysis (Table 2, Fig. 1B). The summary statistics of the deregulated genes obtained after pairwise differential analysis is provided in Table 3. The expression of a selection of genes involved in diverse biological processes (such as cell adhesion, response to oxidative stress, ATPase activity, protein chaperons, lipid metabolism, hatching and tissue regeneration) altered after DU-exposure is displayed in Fig. 2. The gene ontology analysis (GO) was applied to classify the most significantly affected pathways in each condition (Table 4).

**Table 1:** Description of the zebrafish samples collected in the study. Biological replicates are indicated as well as the number of reads, the quality score (Phred score, Q) and the read's length. PF: reads passing the Illumina's chastity filter.

***Sample name***	***Tissu/Stage***	***Organism***	***Genotype***	***Treatment***	***Description***	***Reads***	***Q (PF)***	***Length***
C2cells_r1	2 cells embryos	Danio rerio	Wild type AB	Progeny of adult exposed fish	Two cells embryos obtained from non exposed adult	136,445,062	38.57	51
C2cells_r2	2 cells embryos	Danio rerio	Wild type AB	Progeny of adult exposed fish	Two cells embryos obtained from non exposed adult	84,864,394	38.62	51
C2cells_r4	2 cells embryos	Danio rerio	Wild type AB	Progeny of adult exposed fish	Two cells embryos obtained from non exposed adult	64,705,960	38.62	51
C96hpf_l1	96 hpf larvae	Danio rerio	Wild type AB	Progeny of adult exposed fish	96hpf larvae obtaiend from non exposed adult	74,497,510	35.93	70
C96hpf_l2	96 hpf larvae	Danio rerio	Wild type AB	Progeny of adult exposed fish	96hpf larvae obtaiend from non exposed adult	101,797,850	35.87	70
C96hpf_l3	96 hpf larvae	Danio rerio	Wild type AB	Progeny of adult exposed fish	96hpf larvae obtaiend from non exposed adult	71,463,198	35.88	70
CbrainF_b2	Adult brain	Danio rerio	Wild type AB	20 µg/L depleted uranium	Brain dissected from non exposed adult females	99,758,552	35.71	70
CbrainF_b3	Adult brain	Danio rerio	Wild type AB	20 µg/L depleted uranium	Brain dissected from non exposed adult females	69,241,514	35.71	70
CbrainF_b5	Adult brain	Danio rerio	Wild type AB	20 µg/L depleted uranium	Brain dissected from non exposed adult females	102,185,518	35.7	70
CbrainM_b1	Adult brain	Danio rerio	Wild type AB	20 µg/L depleted uranium	Brain dissected from non exposed adult males	42,244,184	35.71	70
CbrainM_b4	Adult brain	Danio rerio	Wild type AB	20 µg/L depleted uranium	Brain dissected from non exposed adult males	91,270,976	35.73	70
CbrainM_b5	Adult brain	Danio rerio	Wild type AB	20 µg/L depleted uranium	Brain dissected from non exposed adult males	119,455,624	35.71	70
CovaryF_g1	Adult ovary	Danio rerio	Wild type AB	20 µg/L depleted uranium	Ovaries dissected from non exposed adult females	80,198,724	35.72	70
CovaryF_g2	Adult ovary	Danio rerio	Wild type AB	20 µg/L depleted uranium	Ovaries dissected from non exposed adult females	107,238,778	35.66	70
CovaryF_g5	Adult ovary	Danio rerio	Wild type AB	20 µg/L depleted uranium	Ovaries dissected from non exposed adult females	55,595,382	35.62	70
CtestesM_g1	Adult testis	Danio rerio	Wild type AB	20 µg/L depleted uranium	Testies dissected from non exposed adult males	117,865,394	35.68	70
CtestesM_g3	Adult testis	Danio rerio	Wild type AB	20 µg/L depleted uranium	Testies dissected from non exposed adult males	82,404,950	35.67	70
CtestesM_g5	Adult testis	Danio rerio	Wild type AB	20 µg/L depleted uranium	Testies dissected from non exposed adult males	85,837,636	35.66	70
U2cells_r1	2 cells embryos	Danio rerio	Wild type AB	Progeny of adult exposed fish	Two cells embryos obtained from depleted uranium exposed adult	114,044,060	38.15	51
U2cells_r3	2 cells embryos	Danio rerio	Wild type AB	Progeny of adult exposed fish	Two cells embryos obtained from depleted uranium exposed adult	119,056,692	38.34	51
U2cells_r5	2 cells embryos	Danio rerio	Wild type AB	Progeny of adult exposed fish	Two cells embryos obtained from depleted uranium exposed adult	116,943,736	38.31	51
U96hpf_l1	96 hpf larvae	Danio rerio	Wild type AB	Progeny of adult exposed fish	96hpf larvae obtaiend from depleted uranium exposed adult	70,360,064	35.89	70
U96hpf_l2	96 hpf larvae	Danio rerio	Wild type AB	Progeny of adult exposed fish	96hpf larvae obtaiend from depleted uranium exposed adult	79,320,508	35.86	70
U96hpf_l3	96 hpf larvae	Danio rerio	Wild type AB	Progeny of adult exposed fish	96hpf larvae obtaiend from depleted uranium exposed adult	71,471,552	35.88	70
UbrainF_b2	Adult brain	Danio rerio	Wild type AB	20 µg/L depleted uranium	Brain dissected from depleted uranium exposed adult females	67,726,036	35.69	70
UbrainF_b4	Adult brain	Danio rerio	Wild type AB	20 µg/L depleted uranium	Brain dissected from depleted uranium exposed adult females	82,049,972	35.67	70
UbrainF_b5	Adult brain	Danio rerio	Wild type AB	20 µg/L depleted uranium	Brain dissected from depleted uranium exposed adult females	66,539,292	35.62	70
UbrainM_b2	Adult brain	Danio rerio	Wild type AB	20 µg/L depleted uranium	Brain dissected from depleted uranium exposed adult males	98,234,866	35.73	70
UbrainM_b3	Adult brain	Danio rerio	Wild type AB	20 µg/L depleted uranium	Brain dissected from depleted uranium exposed adult males	106,038,446	35.73	70
UbrainM_b5	Adult brain	Danio rerio	Wild type AB	20 µg/L depleted uranium	Brain dissected from depleted uranium exposed adult males	100,296,920	35.71	70
UovaryF_g1	Adult ovary	Danio rerio	Wild type AB	20 µg/L depleted uranium	Ovaries dissected from depleted uranium exposed adult females	89,150,118	35.86	70
UovaryF_g4	Adult ovary	Danio rerio	Wild type AB	20 µg/L depleted uranium	Ovaries dissected from depleted uranium exposed adult females	77,213,772	35.87	70
UtestesM_g1	Adult testis	Danio rerio	Wild type AB	20 µg/L depleted uranium	Testies dissected from depleted uranium exposed adult males	67,017,732	35.88	70
UtestesM_g3	Adult testis	Danio rerio	Wild type AB	20 µg/L depleted uranium	Testies dissected from depleted uranium exposed adult males	65,968,150	35.92	70
UtestesM_g5	Adult testis	Danio rerio	Wild type AB	20 µg/L depleted uranium	Testies dissected from depleted uranium exposed adult males	79,436,904	35.83	70

**Table 2:** List of the 22 samples with the lowest biological variabilities.

***Sample name***	***Tissu*****/*****stage***	***Treatment***
C2cells_r1	2 cells embryos	Progeny of adult exposed fish
C2cells_r2	2 cells embryos	Progeny of adult exposed fish
C2cells_r4	2 cells embryos	Progeny of adult exposed fish
C96hpf_l2	96 hpf larvae	Progeny of adult exposed fish
C96hpf_l3	96 hpf larvae	Progeny of adult exposed fish
CbrainF_b2	Adult brain	20 µg/L depleted uranium
CbrainF_b5	Adult brain	20 µg/L depleted uranium
CovaryF_g1	Adult ovary	20 µg/L depleted uranium
CovaryF_g2	Adult ovary	20 µg/L depleted uranium
CtestesM_g3	Adult testis	20 µg/L depleted uranium
CtestesM_g5	Adult testis	20 µg/L depleted uranium
U2cells_r1	2 cells embryos	Progeny of adult exposed fish
U2cells_r3	2 cells embryos	Progeny of adult exposed fish
U2cells_r5	2 cells embryos	Progeny of adult exposed fish
U96hpf_l2	96 hpf larvae	Progeny of adult exposed fish
U96hpf_l3	96 hpf larvae	Progeny of adult exposed fish
UbrainF_b2	Adult brain	20 µg/L depleted uranium
UbrainF_b5	Adult brain	20 µg/L depleted uranium
UovaryF_g1	Adult ovary	20 µg/L depleted uranium
UovaryF_g4	Adult ovary	20 µg/L depleted uranium
UtestesM_g3	Adult testis	20 µg/L depleted uranium
UtestesM_g5	Adult testis	20 µg/L depleted uranium

**Table 3:** Summary statistics of the differentially expressed genes (threshold FDR<0.01 and fold change>=±2). The number of genes is indicated for each category.

***DU*****/*****Ctrl***	***Up-regulated***	***Down-regulated***	***Total***
***Two-cells***	2482	4106	6588
***96 hpf***	573	62	635
***Brain***	46	1026	1072
***Testis***	132	295	427
***Ovaries***	302	169	471

**Table 4:** Gene ontology analysis. The list provides the top 5 biological pathways impacted by DU in each dataset based on smallest *p*-values.

***Set of genes***	***Gene ontology term***	***p-value***
*Down in DU exposed brain*	Visual perception	2.7E−08
	Sensory perception of light stimulus	2.7E−08
	Cell adhesion	6.3E−07
	Biological adhesion	6.3E−07
	Response to light stimulus	5.1E−06
		
*Up in DU exposed brain*	Response to lipid	2.4E−03
	Response to lipopolysaccharide	2.7E−03
	Ovulation	3.1E−03
	Response to molecule of bacterial origin	3.1E−03
	Response to organic substance	3.5E−03
		
*Down in DU exposed testis*	Cell adhesion	1.0E−04
	Biological adhesion	1.0E−04
	Fin regeneration	3.4E−04
	cGMP biosynthetic process	4.9E−04
	cGMP metabolic process	4.9E−04
		
*Up in DU exposed testis*	Centromere complex assembly	8.2E−04
	Regulation of synapse structure or activation	3.4E−03
	Peptidoglycan biosynthetic process	4.4E−03
	Peptidoglycan-based cell wall biogenesis	4.4E−03
	Protein side chain deglutamylation	4.4E−03
		
*Down in DU exposed ovaries*	Aspartate family amino acid metabolic pr.	4.1E−04
	Endomembrane system organization	4.7E−03
	Androgen biosynthetic process	5.9E−03
	Mitochondrial protein catabolic process	5.9E−03
	Mitophagy by induced vacuole formation	5.9E−03
		
*Up in DU exposed ovaries*	Neural tube development	7.8E−07
	Embryonic appendage morphogenesis	2.6E−06
	Pectoral fin development	4.7E−06
	Fin morphogenesis	5.2E−06
	Appendage morphogenesis	8.2E−06
		
*Down in 2 cells stage*	ncRNA metabolic process	1.6E−14
	Nucleic acid metabolic process	3.6E−14
	Cellular macromolecule metabolic process	3.7E−12
	Organelle organization	2.6E−11
	Heterocycle metabolic process	3.2E−11
		
*Up in 2 cells stage*	Cytoplasmic transport	3.8E−06
	Organelle organization	7.2E−06
	ER to Golgi vesicle-mediated transport	1.1E−05
	Double-strand break repair	1.3E−05
	mRNA processing	1.5E−05
		
*Down in 96hpf larva*	Thyroid hormone generation	2.1E−03
	Thyroid hormone metabolic process	2.1E−03
	Hydrogen peroxide biosynthetic process	2.1E−03
	Circadian rhythm	3.0E−03
	Homophilic cell adhesion via plasma memb.	3.4E−03
		
*Up in 96hpf larva*	Oxidation-reduction process	6.6E−22
	Single-organism metabolic process	1.4E−20
	Small molecule metabolic process	1.4E−16
	Organic acid metabolic process	9.9E−15
	Lipid metabolic process	1.3E−14

## 2 Experimental design, materials and methods

### 2.1 Exposure to depleted uranium and fish maintenance

Adult wild type zebrafish of the AB genetic background (30 females and 30 males, 6–9 months of age) were obtained from Amagen (Gif-sur-Yvette, France) and acclimated for 3 weeks in 30 L glass tanks containing synthetic soft water (CaCl_2_[2H_2_O] 42.49 mg/L, MgCl_2_[6H_2_O] 19.30 mg/L, MgSO_4_[7H_2_O] 24.65 mg/L, Na_2_CO_3_ 0.78 mg/L, KCl 11.33 mg/L, and NaNO_3_ 26.35 mg/L) and oxygenated by bubbling with air. The density was maintained to one fish per litre. Housing conditions were maintained through the acclimatization phase and during DU exposure to: 28 °C±1 °C, pH to 6.5±0.1 and under a day light cycle of 14 h/10 h (day/night). Fish were fed once a day with 24hpf *Artemia salina nauplii* (JBL, Herblay, France) and twice a day with standard fish flakes (Tetramin, Melle, Germany). Males and females were kept separated and crossed once a week during the 3 weeks of acclimatization. Fish were then exposed to 20 µg/L DU (UO_2_(NO_3_)_2_ – 6H_2_O, Sigma, Lezennes, France). DU concentration was checked several times per day by ICP-MS (7500Cx spectrometer, Agilent) in technical triplicates. The actual DU concentration in the tanks over the 10 days was 15.5±2.5 μg/L DU for the males and 17.4±3 μg/L for the females. After 6 days of exposure to DU, all males and all females were mated in clean water for 4 h. Adult fish were replaced in DU contaminated water after the mating for 4 more days (10 days in total). Embryos were grown in clean water for 4 days at 28 °C in incubators (TC series, Aqualytics). No death, behavioural differences or sign of suffering were observed in the DU-exposed fish group as compared to controls. Measurement of body mass and length didn’t reveal any difference between the exposed and control group. All fish were killed by immersion in ice cold water at the end of the experiment and tissue dissected under the binocular (Leica, France).

### 2.2 Extraction of total RNA

Total RNA was extracted with the Absolutely RNA Miniprep kit (Agilent) according to manufacturer's recommendations. Single adult tissue was used for the extraction. Pools of three larvae were used at the four-days stage and pools of 50 embryos at two-cells stage. RNA integrity was checked by loading about 100 ng total RNA on a RNA6000 Nanochip using an Agilent 2100 Bioanalyser (Agilent Technologies). Samples showed no sign of degradation (RNA index number>8).

### 2.3 Library preparation, quality control

Total RNA (1 µg) was subjected to two rounds of poly(A) RNA selection using poly-dT coated magnetic beads using the strand-oriented TruSeq mRNA kit v2 (Illumina) following manufacturer's protocol. First-strand cDNA synthesis was performed with the Superscript II (Thermo Fisher) using random hexamer primers, cDNA fragments subjected to end-repair, dA-tailing, and finally ligated to adapters. Libraries were amplified by 12 cycles of PCR. The quality and concentration of the sequencing libraries were checked on a DNA1000 chip (2100 Bioanalyser, Agilent Technologies), multiplexed at 8 pM and sequenced in the paired-end mode on a HiSeq. 1500 device (Illumina) to generate 2×51 or 2×70 bp paired-end reads (Table 1). Base calling was performed using RTA v.1.13 (Illumina). Bad quality reads were filtered out with *trimgalore* using the option –q 30 and –paired.

### 2.4 Data analysis

Mapping of filtered reads was performed on the Zv10 indexed genome generated with the exon-exon information from Ensembl (release 85) with RNA-STAR [3] using the options –alignIntronMax 1000000 –alignMatesGapMax 1000000 –alignIntronMin 20 –outFilterMultimapNmax 20 –outWigStrand Stranded –quantMode TranscriptomeSAM GeneCounts. Quantification and normalization of the mapped reads at the level of gene model were performed with DESeq. 2 [4]. Adjusted *p*-values (False Discovery Rate, FDR)<0.01 and fold-change>=±2 were used to detect significant differential gene expression. Gene Ontology (GO) analysis was performed with the R package *TopGO* using the *Danio rerio* annotations from Ensembl (release 85).

## Ethic approval

All experiments were made in accordance with the French animal protection standards and were approved by the Animal User and Ethical Committee at the IRSN (committee 81).
